# Gut microbiota diversity is prognostic in metastatic hormone receptor‐positive breast cancer patients receiving chemotherapy and immunotherapy

**DOI:** 10.1002/1878-0261.70117

**Published:** 2025-08-25

**Authors:** Andreas Ullern, Kristian Holm, Nikolai Kragøe Andresen, Andreas Hagen Røssevold, Corinna Bang, Bjørn Naume, Johannes R. Hov, Jon Amund Kyte

**Affiliations:** ^1^ Department of Clinical Cancer Research Oslo University Hospital Oslo Norway; ^2^ Department of Cancer Immunology, Institute for Cancer Research Oslo University Hospital Norway; ^3^ Institute of Clinical Medicine University of Oslo Norway; ^4^ Research Institute of Internal Medicine Oslo University Hospital Rikshospitalet Oslo Norway; ^5^ Norwegian PSC Research Center, Department of Transplantation Medicine Oslo University Hospital Oslo Norway; ^6^ Institute of Clinical Molecular Biology Christian‐Albrechts‐University of Kiel Germany; ^7^ Department of Oncology Oslo University Hospital Oslo Norway; ^8^ Section of Gastroenterology, Department of Transplantation Medicine Oslo University Hospital Oslo Norway; ^9^ Faculty of Health Sciences Oslo Metropolitan University Oslo Norway

**Keywords:** alpha diversity, gut microbiota, hormone receptor‐positive breast cancer, immunotherapy

## Abstract

Immune checkpoint blockade (ICB) is standard treatment in several cancer types, despite not being proven efficacious in metastatic hormone receptor‐positive breast cancer (HR+ mBC). The gut microbiota is associated with patient outcome and toxicity from cancer therapy, although limited data are available for breast cancer. In the randomized phase 2b trial ICON, immunomodulating chemotherapy was investigated in combination with dual ICB in HR+ mBC. To determine whether gut microbiota could inform prognosis, we performed 16S (V3‐V4) rRNA sequencing on fecal samples collected at baseline and after 8 weeks of study treatment. We showed that high alpha diversity before treatment was associated with prolonged progression‐free survival (PFS; primary trial endpoint) and overall survival. Alpha diversity was lower in patients with prior chemotherapy in the metastatic setting. However, alpha diversity remained significantly associated with PFS after correcting for prior chemotherapy and other factors in bivariate analyses. High‐grade immune‐related toxicity was also associated with high alpha diversity. These findings suggest that high alpha diversity should be further investigated as a positive prognostic factor in HR+ mBC and approaches to increase alpha diversity could potentially improve clinical outcome.

## Graphical abstract

In this exploratory study, we investigated the relationship between the gut microbiota and outcome in patients with metastatic hormone receptor‐positive breast cancer, treated in a randomized clinical trial with chemotherapy alone or chemotherapy in combination with immune checkpoint blockade. Our data suggest a prognostic role of alpha diversity in this population.

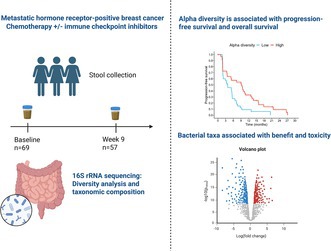

**Abbreviations**
ANCOM‐BC: analysis of compositions of microbiomes with bias correction
ASV: amplicon sequence variant
BC: breast cancer
Faith's PD: Faith's phylogenetic diversity
HR+: hormone receptor‐positive
ICB: immune checkpoint blockade
IrAE: immune‐related adverse event
OS: overall survival
PFS: progression‐free survival

## 1 Introduction

Breast cancer is classically categorized based on the expression of hormone receptors and human epidermal growth factor receptor 2 (HER2) in tumor cells. Hormone receptor‐positive breast cancer (HR+ BC) is the most common subtype of BC, and metastatic disease (mBC) remains incurable. The gut microbiota, the intestinal ecosystem of microbes, interacts with the host, and influences many aspects of human health, such as the development of the host immune system, protection against pathogens, maintaining barrier integrity, and regulation of host metabolism [1]. The gut microbiota has emerged as a possible biomarker in cancer, predicting both response to treatment and toxicity—and a potential target for interventions to improve outcomes [2]. The interaction between the gut microbiota and immune checkpoint blockade (ICB) has gained particular interest. ICB has transformed the treatment landscape for many cancer forms and shown effects against early‐stage triple‐negative BC (TNBC) and metastatic triple‐negative breast cancer (mTNBC) expressing programmed death‐ligand 1 (PD‐L1) [3, 4]. Recently, promising results have been published in early HR+ BC [5, 6], but there are limited data and no documented efficacy from ICB in metastatic disease [7].

Most studies exploring the relationship between the gut microbiota and ICB have been conducted in cancer forms in which ICB is an effective therapy, such as melanoma, non‐small cell lung cancer, and renal cancer [8]. However, substantial heterogeneity has been observed across different study cohorts, limiting the generalizability of gut microbiota biomarkers [8, 9]. Furthermore, these results may not be transferable to HR+ BC, which has been considered far less immunogenic [10]. In HR+ BC, some studies have assessed the gut microbiota in patients receiving chemotherapy [11, 12, 13, 14, 15]. Recently, gut and oral microbiota data were reported from a single‐arm study of HR+ mBC treated with pembrolizumab and eribulin [16]. In addition, we have previously shown that a high gut microbiota alpha diversity was associated with improved clinical outcome in mTNBC patients receiving chemo‐immunotherapy and possibly with benefit from PD‐L1 blockade [17].

In the randomized phase 2b ICON trial, we evaluated the combination of anthracycline‐based chemotherapy and dual ICB in patients with HR+ mBC [18]. A selected chemotherapy backbone with immunomodulating properties was applied and combined with antibodies blocking CTLA4 (ipilimumab) and PD1 (nivolumab). We found that the concomitant addition of ICB to chemotherapy did not improve efficacy [19]. Still, clinical responses were obtained in a study cohort receiving ipilimumab and nivolumab sequential to the study chemotherapy.

Here, we investigated the impact of the gut microbiota in HR+ mBC by analyzing fecal samples from the ICON trial. We assessed the association between the diversity and composition of the gut microbiota and clinical response, as assessed by progression‐free survival, overall survival, and clinical benefit. We furthermore evaluated the relationship between the gut microbiota and high‐grade immune‐related adverse events and the kinetics of alpha diversity during study treatment.

## 2 Materials and methods

### 2.1 Study design and participants

All patients were enrolled in the randomized, open‐label, phase 2b ICON trial (NCT03409198) [18, 19]. In ICON, patients with HR+ mBC starting first‐/second‐line chemotherapy were randomized 2:3 to chemotherapy alone (chemo‐only; *n* = 33) or chemotherapy in combination with concomitant dual ICB (ipilimumab and nivolumab; immune‐chemo; *n* = 50). The chemotherapy regimen was the same in both arms and consisted of pegylated liposomal doxorubicin (PLD) 20 mg·m^−2^ i.v. every second week and cyclophosphamide 50 mg p.o. daily in every second 2‐week cycle. ICB dosing was ipilimumab 1 mg/kg every sixth week and nivolumab 240 mg every second week.

Patients were enrolled in five hospitals in Norway (Oslo University Hospital, Stavanger University Hospital and Sørlandet Hospital) and Belgium (Institut Jules Bordet and CHU UCL Namur) between February 2018 and November 2020. Eligible patients were 18 years or older with metastatic estrogen receptor‐positive, HER2‐negative breast cancer, measurable disease by Response Evaluation Criteria In Solid Tumors V.1.1 (RECIST V.1.1), Eastern Cooperative Oncology Group (ECOG) performance status 0–1, and a maximum of one previous line of chemotherapy for metastatic disease. Study approval was obtained by the Norwegian Medicines Agency, the Belgian Federal Agency for Medicines and Health Products, and the regional committees for medical research ethics (Norwegian reference no.: 2017/1283, Belgian reference no.: 19021). All patients provided written informed consent. The consent and approval included the analyses presented in the present article, and the trial was conducted in adherence to the Declaration of Helsinki.

The primary endpoints in ICON were safety and progression‐free survival (PFS). Secondary/exploratory endpoints included overall survival (OS), clinical benefit rate, and biomarkers for clinical efficacy and toxicity. To be eligible for microbiota profiling, patients had to deliver a fecal sample at baseline and receive any study drug.

### 2.2 Sample collection

Sample collection, DNA extraction, sequencing, and bioinformatics were performed as previously published [17]. Stool samples were submitted at baseline (prior to beginning any study treatment) and at Week 9 (after 8 weeks of study treatment). The samples were collected in 15‐mL sterile containers, kept by the patients at 4 °C, and subsequently frozen at −80 °C within 24 h.

### 2.3 Fecal DNA extraction and 16S sequencing

Briefly, DNA was extracted employing the QIAamp Fast DNA stool mini kit automated on the QIAcube with prior bead‐beating to ensure proper cell wall lysis of bacteria. The V3 and V4 variable regions of the 16S rRNA gene were amplified using 3 μL of DNA and the primer pair 357F‐806R in a dual‐barcoding approach in line with Kozich et al. [20]. PCR products were verified using electrophoresis in agarose gel, normalized using the SequalPrep Normalization Plate Kit (Thermo Fischer Scientific, Waltham, MA, USA), pooled equimolarly, and sequenced on the Illumina MiSeq v3 2 × 300bp (Illumina Inc., San Diego, CA, USA).

### 2.4 Sequence processing and bioinformatics

Demultiplexing of samples after sequencing was based on 0 mismatches in the barcode sequences. Paired‐end reads were trimmed for primers with cutadapt version 4.0 [21] (parameters: ‐e 0.1 ‐u 1 ‐U 1 ‐‐discard‐untrimmed ‐m 250); then, quality trimmed and merged using bbmerge version 38.90 [22] (parameters: qtrim = r trimq = 15 maxlength = 500 mininsert = 350). The merged contigs were trimmed to 400 bp and denoised to amplicon sequence variants (ASVs) with deblur [23] in qiime2 version 2022.2 [24]. Taxonomic classification of ASVs was performed based on RESCRIPt [25] in qiime2, applying a naïve Bayes classifier [26] trained on the V3‐V4 region of the nonredundant NR99 subset of the Silva database version 138 [27].

ASVs from mitochondria, chloroplast, or lacking taxonomic annotation on order level were removed, and a de‐novo phylogenetic tree was built in qiime2 based on the remaining ASVs. To control for uneven sequencing depths, samples were rarefied (subsampled without replacement) to an even depth of 6411 counts per sample. All analyses except differential abundance were performed on this rarefied dataset. Alpha diversity metrics (Shannon diversity, Faith's phylogenetic diversity (PD), and observed ASVs) were calculated in qiime2.

### 2.5 Statistical analysis

The following endpoints were chosen for the microbiota analysis: PFS (the time from randomization until objective disease progression or death), OS (the time from randomization until death), clinical benefit (proportion of patients with an objective tumor response or with stable disease lasting ≥24 weeks), and high‐grade immune‐related adverse events (irAEs). IrAEs were scored according to the Common Terminology Criteria for Adverse Events (CTCAE) version 4.0, and high‐grade irAEs were defined as grade ≥3.

Kaplan–Meier survival curves were produced and compared by log‐rank test. Reverse Kaplan–Meier was applied to estimate the median follow‐up time, with censoring for overall survival as the event. The Cox proportional hazards model was used to estimate hazard ratios (HR) with 95% confidence intervals (CI). Patient and disease characteristics, including alpha diversity metrics, were analyzed by univariate Cox proportional hazards model for PFS and OS. Covariates with *P* < 0.1 from the univariate analysis were considered in the bivariate analyses. R packages survival [28] and survminer [29] were applied in time‐to‐event analyses.

Analysis of composition of microbiomes with bias correction 2 (ANCOM‐BC2) [30] was used for differential abundance analysis. ANCOM‐BC2 was performed on the nonrarefied dataset at genus level, and only genera present in at least 10% of all samples were included. Alpha diversity was compared between groups by Wilcoxon rank‐sum test. Longitudinal analysis of alpha diversity was done by Wilcoxon signed‐rank test. All *P* values given are two‐tailed. *P* values <0.05 were considered statistically significant. Analyses were performed in R [31] using the packages phyloseq [32], qiime2r [33], and ggplot2 [34].

## 3 Results

### 3.1 Patient characteristics

**Fig. 1:**
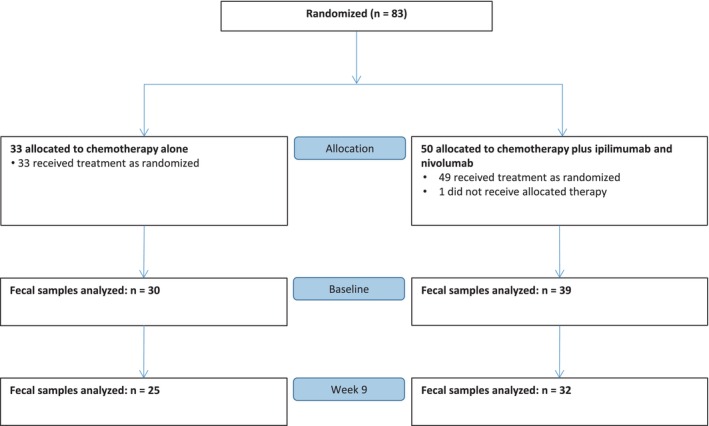
Stool collection in the ICON trial. Stool samples were collected before the start of study treatment (baseline, *n* = 69) and after 8 weeks of treatment (Week 9, *n* = 57) in both treatment arms.

Eighty‐three patients were randomized in the ICON trial; 33 started treatment in the chemo‐only arm and 49 started treatment in the immune‐chemo arm (Fig. 1). Baseline fecal samples were provided by 30 patients (91%) in the chemo‐only arm and 39 patients (80%) in the immune‐chemo arm. Paired samples (baseline + Week 9) were available for 25 patients in the chemo‐only arm and 32 patients in the immune‐chemo arm. Median follow‐up time was 64.6 months.

Baseline patient characteristics for this cohort are provided in Table S1. The ICON trial was negative for the primary efficacy endpoint PFS and the secondary endpoint OS. For patients with fecal samples at baseline, there was no significant difference in PFS (HR 0.81, 95% CI 0.49–1.33) or OS (HR 1.02, 95% CI 0.62–1.68) between the study arms (Fig. S1), which is in concordance with the full analysis set population [19]. Clinical benefit rate was 47% (14/30) in the chemo‐only arm and 62% (24/39) in the immune‐chemo arm. Baseline alpha diversity did not differ significantly between the two study arms (data not shown). Given the similar features in the study groups and no effect of the intervention, a joint analysis of all participants was chosen as the primary analysis, followed by secondary analyses in each treatment arm.

### 3.2 High microbiota diversity was associated with prolonged PFS and OS

**Fig. 2:**
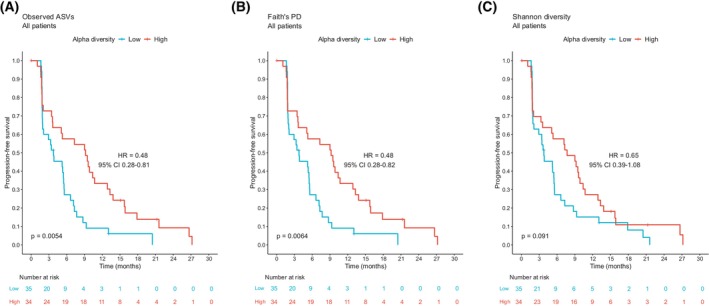
Progression‐free survival by baseline alpha diversity in all patients. Kaplan–Meier plots of progression‐free survival in patients (*n* = 69) with high (> median) and low (≤ median) baseline alpha diversity. Different alpha diversity metrics applied: (A) Observed ASVs, (B) Faith's PD, and (C) Shannon diversity. Hazard ratios with 95% confidence intervals were obtained from the Cox proportional hazards model. *P* values were calculated by the log‐rank method. ASV, amplicon sequence variant; CI, confidence interval; Faith's PD, Faith's phylogenetic diversity; HR, hazard ratio.

Alpha diversity represents the microbiota diversity within a single fecal sample and can be measured by different metrics reflecting richness and/or evenness [35]. We assessed the association between pretreatment (baseline) alpha diversity and PFS and OS, employing three alpha diversity metrics (Observed ASVs, Faith's PD, Shannon diversity). The patients were divided into low and high alpha diversity groups employing the median value as a cutoff. Patients with a high baseline alpha diversity had prolonged PFS compared to those with low alpha diversity, evaluated both with observed ASVs (HR 0.48, 95% CI 0.28–0.81; *P* = 0.0054; Fig. 2A) and Faith's PD (HR 0.48, 95% CI 0.28–0.82; *P* = 0.0064; Fig. 2B), while the association was not statistically significant for Shannon diversity (Fig. 2C). Next, we analyzed the two treatment arms separately. The association between high diversity and prolonged PFS was seen in both treatment arms and using all diversity metrics, but the hazard ratios were lowest and the associations statistically significant only in the chemo‐only arm, for observed ASVs (HR 0.32; *P* = 0.0092, Fig. S2a) and Faith's PD (HR 0.32; *P* = 0.0092, Fig. S2b). Faith's PD and observed ASVs categorized patients likewise and seemed highly correlated, while dichotomization of Shannon gave similar, but slightly different results.

**Fig. 3:**
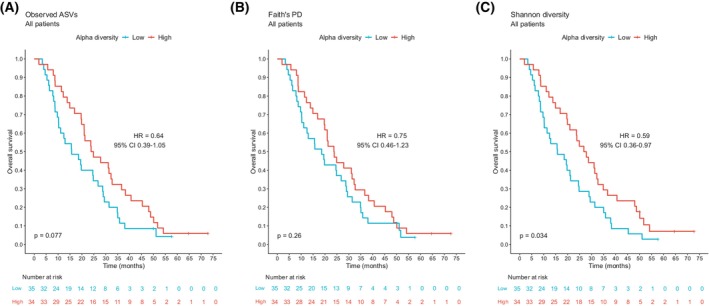
Overall survival by baseline alpha diversity in all patients. Kaplan–Meier plots of overall survival in patients (*n* = 69) with high (> median) and low (≤ median) baseline alpha diversity. Different alpha diversity metrics applied: (A) Observed ASVs, (B) Faith's PD, and (C) Shannon diversity. Hazard ratios and 95% confidence intervals were obtained from the Cox proportional hazards model. *P* values were calculated by the log‐rank method. ASV, amplicon sequence variant; CI, confidence interval; Faith's PD, Faith's phylogenetic diversity; HR, hazard ratio.

High alpha diversity was also associated with prolonged overall survival (Fig. 3). All three alpha diversity metrics yielded similar effect estimates, though statistically significant only for Shannon (HR 0.59, 95% CI 0.36–0.97; *P* = 0.034; Fig. 3C). When analyzing the treatment arms separately, the Kaplan–Meier curves suggested that the relationship between alpha diversity and OS was mainly driven by the immune‐chemo arm (Fig. S3).

### 3.3 Univariate and bivariate analyses of PFS and OS

Univariate Cox proportional hazards analysis for PFS is shown in Table S2. The unadjusted univariate model included alpha diversity metrics as well as 11 clinical variables and was performed on all patients, regardless of study arm. Previous chemotherapy in the metastatic setting was the only clinical variable to be significantly linked to PFS (*P* = 0.014), whereas body mass index and bone metastases had *P* values <0.1 and were also included in the bivariate analyses. Alpha diversity was lower in patients who had received prior chemotherapy in the metastatic setting compared with those that had not (Fig. S4). However, a high alpha diversity remained statistically significant in the bivariate model after controlling for each of the three covariates (Table S3). This applied to both observed ASVs and Faith's PD.

Univariate analysis for the same variables was performed for OS (Table S4). Again, previous chemotherapy in the metastatic setting (*P* = 0.003) was the only significant clinical variable. The number of metastatic sites (>3 sites) had a *P* value <0.1 and was included in bivariate analyses, which are shown in Table S5. The analyses showed that a high Shannon diversity index was borderline significant after controlling for previous metastatic chemotherapy (*P* = 0.082) or the number of metastatic sites (*P* = 0.063).

### 3.4 Association between individual gut bacteria and clinical benefit in the ICON trial

**Fig. 4:**
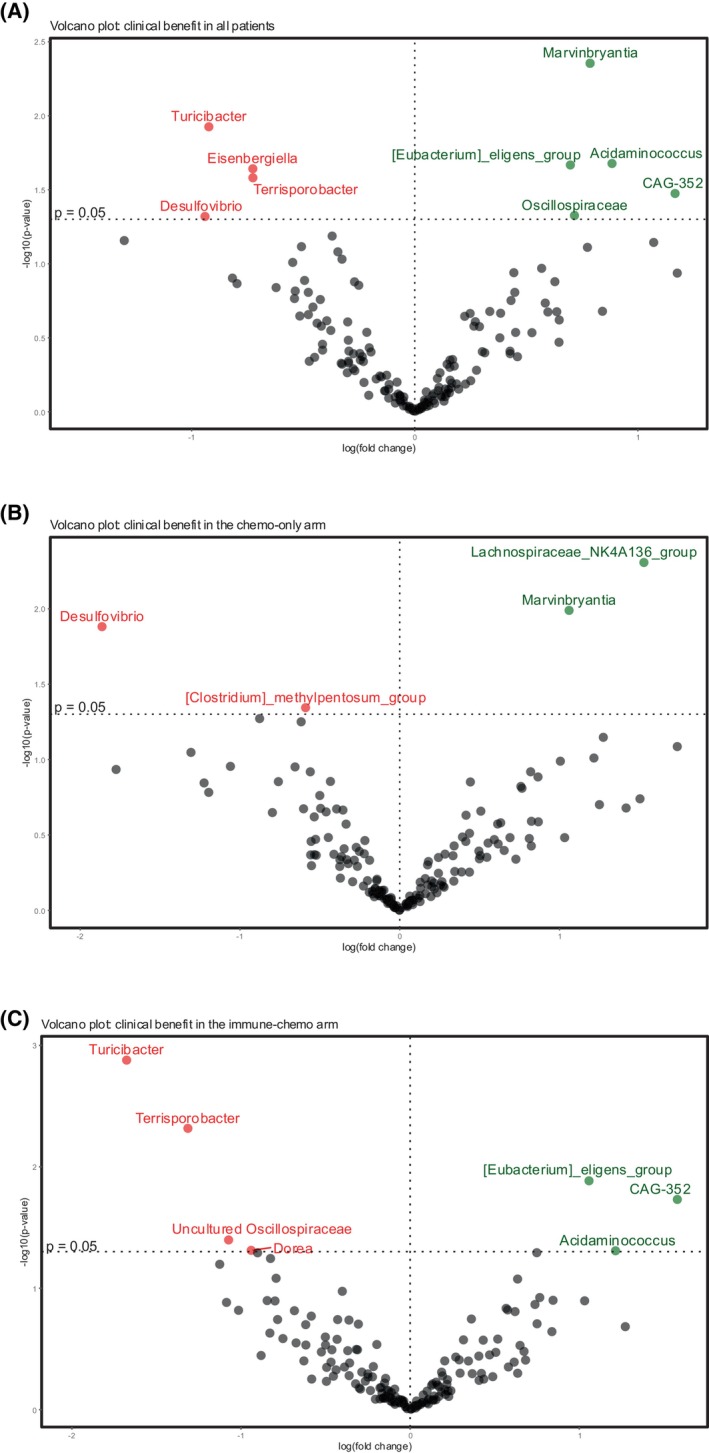
Differential abundance analysis by clinical benefit. Volcano plot from ANCOM‐BC2 showing compositional differences between patients with and without clinical benefit in the ICON trial, in (A) all patients (*n* = 69), (B) the chemo‐only (*n* = 30) and (C) the immune‐chemo arm (*n* = 39). The figures show differentially abundant bacterial taxa in patients with and without clinical benefit. The x‐axis shows effect size, and the y‐axis represents *P* values. Taxa with *P* value <0.05 are displayed above the horizontal dashed line. Taxa enriched in patients with clinical benefit are colored in green, and taxa enriched in patients without clinical benefit are colored in red. The vertical dashed line illustrates log fold change of zero. No taxa were statistically significant after correcting for multiple testing (false discovery rate adjustment in ANCOM‐BC2). ANCOM‐BC2, Analysis of composition of microbiomes with bias correction 2.

For differential abundance analysis, we used ANCOM‐BC2 to identify individual genera associated with the outcome. The categorical endpoint clinical benefit (i.e., an objective tumor response or stable disease lasting ≥24 weeks) was chosen as the covariate of interest. Among all patients, we identified *Marvinbryantia, Acidaminococcus, Eubacterium eligens* group, *CAG‐352*, and *Oscillospiraceae* family as significantly overrepresented in patients with clinical benefit. *Turicibacter, Eisenbergiella, Terrisporobacter*, and *Desulfovibrio* were enriched in those without clinical benefit (Fig. 4A).

When assessing the two treatment arms separately, we observed that patients with clinical benefit in the chemo‐only arm were significantly enriched in *Lachnospiraceae NK4A136* group and *Marvinbryantia*, whereas patients without clinical benefit were enriched in *Desulfovibrio* and *Clostridium methylpentosum* group (Fig. 4B). In the immune‐chemo arm, *Eubacterium eligens* group, *CAG‐352*, and *Acidaminococcus* were significantly overrepresented in patients with clinical benefit, while *Turicibacter, Terrisporobacter*, an unnamed uncultured genus of the *Oscillospiraceae* family, and *Dorea* were observed in those without clinical benefit (Fig. 4C). No single genus was statistically significant after correction for multiple testing in ANCOM‐BC2.

### 3.5 Gut microbial associations with high‐grade irAEs

**Fig. 5:**
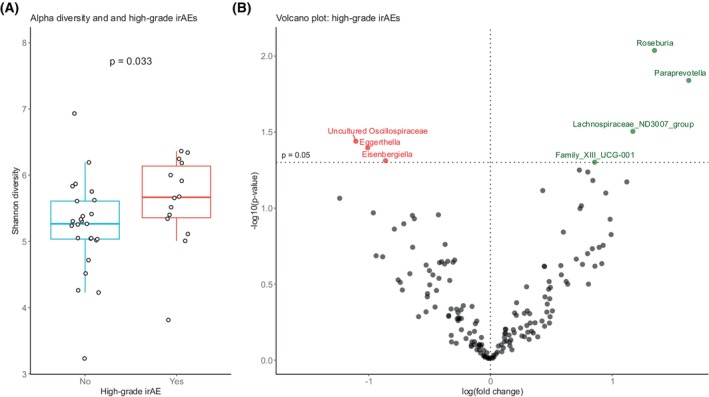
Associations of gut microbiota and high‐grade immune‐related adverse events. (A) Shannon diversity in patients with (*n =* 14) and without (*n* = 25) high‐grade (grade ≥3) immune‐related adverse events (irAEs). The box plot extends from the first to the third quartile. The middle line represents the median, and the whiskers extend to the most extreme points within 1.5 × IQR. Each dot represents a sample. *P* value calculated by Wilcoxon rank‐sum test. (B) Volcano plot based on ANCOM‐BC2. The plot visualizes different abundant bacterial taxa in patients with (*n =* 14) and without (*n* = 25) high‐grade irAEs in the ICON study. The x‐axis represents effect size, and the y‐axis represents *P* values. Taxa with *P* value <0.05 are presented above the horizontal dashed line. Taxa enriched in patients with high‐grade irAEs are colored in green, and taxa enriched in patients without high‐grade irAEs are colored in red. No taxa were statistically significant after correcting for multiple testing (false discovery rate adjustment in ANCOM‐BC2). ANCOM‐BC2, Analysis of composition of microbiome with bias correction 2; irAE, immune‐related adverse events.

A high rate of irAEs was observed in the immune‐chemo arm. Intriguingly, high‐grade irAEs were associated with prolonged PFS [19]. For patients with available baseline stool samples, high‐grade (grade ≥3) irAEs were observed in 36% (14/39) in the immune‐chemo arm. We investigated whether high‐grade irAEs were associated with microbiota alpha diversity. The results showed that patients who developed high‐grade irAEs had significantly higher Shannon diversity at baseline, compared with patients without high‐grade irAEs (*P* = 0.033, Fig. 5A). Other diversity metrics displayed similar trends, but were not statistically significant (Fig. S5a,b).

Differential abundance analysis by ANCOM‐BC2 revealed four taxa associated with high‐grade irAEs. The genus most significantly associated with high‐grade irAEs in our analysis was *Roseburia. Paraprevotella, Lachnospiraceae ND3007* group, and *Family X11 UCG‐001* were also enriched in patients with irAEs grade ≥3. Uncultured genus of the *Oscillospiraceae* family, *Eggerthella*, and *Eisenbergiella* were overrepresented in patients without severe irAEs (Fig. 5B).

### 3.6 Longitudinal analysis of alpha diversity during treatment

**Fig. 6:**
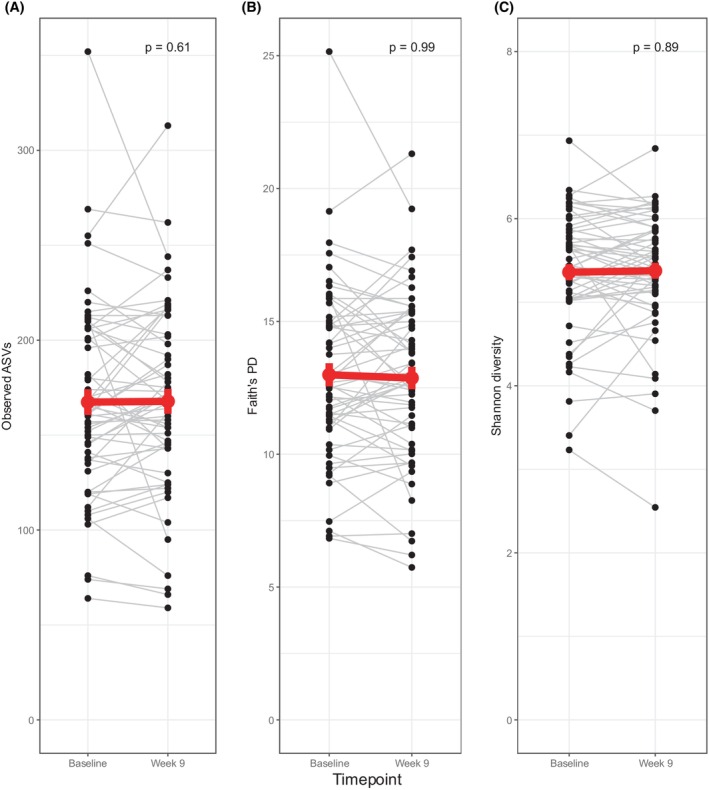
Longitudinal analysis of alpha diversity. Alpha diversity in the course of study treatment for all patients with available paired stool samples (baseline and Week 9, *n =* 57). Different alpha diversity metrics applied: (A) Observed ASVs (B) Faith's PD, and (C) Shannon diversity. *P* values were calculated using the Wilcoxon signed‐rank test. The red point represents the mean alpha diversity at each time point, and the error bars represent the standard error of the mean. The red line represents the change of the mean alpha diversity value from baseline to Week 9. ASV, amplicon sequence variant; Faith's PD, Faith's phylogenetic diversity.

We assessed changes in alpha diversity during the course of study treatment by pairwise comparison of baseline samples to Week 9 samples in all patients providing stool samples at both timepoints (*n* = 57). The alpha diversity remained stable throughout the first 2 months of treatment (Fig. 6A–C). Similarly, there was no significant change in alpha diversity in any of the treatment arms (Fig. S6a–c). Eight patients with available paired fecal samples received antibiotics during the first 8 weeks. Alpha diversity did not change significantly in these patients between baseline and Week 9 (Fig. S7a–c).

## 4 Discussion

In this study, we found that gut microbiota alpha diversity was correlated with clinical outcome in HR+ mBC patients treated with chemotherapy alone or chemotherapy in combination with dual ICB. High alpha diversity was associated with both prolonged PFS and OS. Previous chemotherapy in the metastatic setting was associated with outcome and reduced alpha diversity, while the interventions in the present study did not change the microbiota *per se*. High alpha diversity was also linked to more high‐grade irAEs. Overall, our data suggest that high baseline alpha diversity is a positive prognostic factor in this patient population.

High alpha diversity was consistently associated with a favorable outcome in this population of HR+ mBC. The effect was most pronounced as measured by PFS but also detectable when assessed by OS. There was a notable difference in the strength by which the different diversity metrics were associated with outcome, with observed ASVs and Faith's PD being significantly associated with PFS and Shannon diversity with OS. These metrics represent different features of diversity like richness, evenness, and phylogenetic relationship, but are closely correlated and in this context likely represent the same biological phenomenon. When analyzing each treatment arm separately, statistically significant associations were demonstrated in the chemo‐only arm for PFS and in the immune‐chemo arm for OS. However, the sample size in each study arm was low, and this apparent discordance between the study arms should be interpreted with caution. Importantly, high alpha diversity is often considered a global marker of a healthy gut microbiota [36].

The present results are in line with our previously reported association between the gut microbiota and outcome in mTNBC patients receiving chemo‐immunotherapy in the ALICE trial [17]. In ALICE, we applied the same immunomodulating chemotherapy as in ICON but combined with atezolizumab (aPD‐L1) [37]. A similar correlation between high baseline alpha diversity and prolonged PFS was found in the ALICE study, as reported herein for ICON. In contrast to ICON, the addition of ICB to chemotherapy did improve PFS in ALICE [38]. Moreover, a high Faith's PD was predictive of ICB benefit in the ALICE trial [17]. Other studies in breast cancer have reported conflicting results on the relationship between alpha diversity and outcome. In the CALADRIO study on 28 patients, Shannon diversity was not shown to be associated with clinical benefit from pembrolizumab and eribulin in HR+ mBC [16]. A small study (*n* = 23) in early BC of various subtypes found a correlation between alpha diversity and response to neoadjuvant therapy [12], and a similar finding was made in a study of neoadjuvant therapy against early HER2+ BC [39]. A larger study found no significant relationship between alpha diversity and pathological complete response to neoadjuvant chemotherapy in early breast cancer [14]. Importantly, the clinical endpoints in these studies were response (clinical or pathological) or clinical benefit from treatment, and not time‐to‐event outcomes like PFS or OS. Also, different sequencing methods and alpha diversity metrics have been applied. Furthermore, the gut microbiota may differ according to BC type, stage, grade, menopausal status, and other factors [40]. This underscores that care should be taken when comparing different studies, even in the same cancer form.

The gut microbiota may influence the outcome of cancer therapy, but treatment also induces changes in the gut microbiota [41]. In the ALICE study, we found that the gut alpha diversity in mTNBC patients was reduced after 8 weeks of treatment with chemotherapy and ICB. Similar observations have been reported in other cancer forms [42]. By contrast, in the present study, alpha diversity was stable during the first 8 weeks of treatment in both treatment arms. As the chemotherapy regimen was the same in ALICE and ICON, this discrepancy could mirror the differences in biology between mTNBC and HR+ mBC. For patients providing fecal samples at baseline, PFS was 3.8 months in ALICE (mTNBC) and 5.2 months in ICON (HR+ mBC). It is possible that the reduction in alpha diversity observed in ALICE during the first 8 weeks was not a consequence of chemotherapy treatment but rather reflected deterioration of health. In the present study (ICON), alpha diversity was lower in patients who had received prior chemotherapy in the metastatic setting. This may suggest a chemotherapy‐induced perturbation of diversity but could also reflect more advanced disease.

Treatment with ipi/nivo plus chemotherapy was associated with a high rate of irAEs in the ICON trial. A previous landmark analysis demonstrated prolonged PFS in patients who developed high‐grade (grade ≥3) irAEs within the first 4 months in the trial [19]. Interestingly, the most enriched genus in patients with high‐grade irAEs was *Roseburia*, belonging to the Lachnospiraceae family. *Roseburia* is a known producer of the short‐chain fatty acid butyrate, which exerts a variety of functions in the immune system [43], and has been associated with response to ICB across different cancer forms [8]. Others have also found a strong association between *Lachnospiraceae* and irAEs [44]. In our study, moreover, Shannon diversity was significantly higher in patients with high‐grade irAEs compared with those without serious toxicity from ICB. These findings would be consistent with the notion that patients with high‐grade irAEs possessed a healthy gut microbiota, permitting immune activation after ICB and causing irAEs, and possibly prolonged PFS in a subgroup of patients.

There are several limitations to our study. First, the sample size is a limitation, in particular in each of the study arms. Second, the analyses are explorative; independent studies are required to validate our findings. Third, the generalization of findings in microbiota studies is prone to differences in diet and normal bacterial flora between different countries. Various host factors have been associated with alpha diversity, such as BMI, weight, blood pressure, vegetable consumption, and physical activity [45]. Antibiotics represent a possibly confounding factor, as they are more extensively used in patients in a poorer condition and have been reported to impact alpha diversity and to be associated with reduced efficacy of ICB in other cancer forms [46]. In ICON, antibiotic use was recorded for the last 30 days before enrolment and throughout the study. We did not adjust for antibiotic exposure before study enrolment, as this was only recorded in two patients. Of note, alpha diversity was not reduced in the eight patients who received antibiotics between baseline and Week 9.

## 5 Conclusions

In conclusion, our study showed high alpha diversity to be associated with improved PFS and OS in HR+ mBC treated with chemotherapy and ICB. The data indicate a prognostic value of alpha diversity in this patient population. We also found that alpha diversity was associated with high‐grade irAEs, which were in turn associated with prolonged PFS in the immune‐chemo arm. It remains to be seen if gut microbiota interventions, such as diet, probiotics, or fecal microbiota transplantation, may improve outcomes in BC patients. However, our findings suggest that strategies to increase alpha diversity could be pursued, while also pointing to a risk of immune‐related side effects. Future microbiota research in cancer should aim to improve the understanding of involved mechanisms, validate alpha diversity and other candidate biomarkers, and evaluate microbiota interventions.

## Conflict of interest

JAK has, in the last 5 years, received research support from Bristol Myers Squibb, F. Hoffmann‐La Roche, NanoString, and NEC OncoImmunity and has previously received advisory board/lecture honoraria from pharmaceutical companies, including Bristol Myers Squibb. JRH has received grants from Biogen and lecture honoraria from Amgen, Roche, and Novartis. The other authors declare no conflicts of interest.

## Author contributions

JAK was the coordinating investigator and medical monitor of the ICON trial and was responsible for study design and acquisition of funding and approvals. AHR and NKA were investigators at Oslo University Hospital and medical monitors for the other sites. JAK, AHR, and NKA contributed to patient recruitment and data collection. JAK, JRH, and AU designed this study. CB was responsible for stool sample processing and 16S rRNA gene sequencing. AU, AHR, and KH analyzed the data. JAK, AU, AHR, NKA, KH, JRH, and BN interpreted the data. JAK and AU wrote the manuscript, with contributions from all authors. All authors read and approved the manuscript.

## Supporting information


**Fig. S1.** Kaplan–Meier plots of progression‐free survival (a) and overall survival (b) for patients included in the microbiota analysis in ICON.
**Fig. S2.** Progression‐free survival by baseline alpha diversity stratified by treatment arm.
**Fig. S3.** Overall survival by baseline alpha diversity stratified by treatment arm.
**Fig. S4.** Associations between alpha diversity and line of metastatic chemotherapy.
**Fig. S5.** Associations between alpha diversity and high‐grade immune‐related adverse events.
**Fig. S6.** Longitudinal analysis of alpha diversity stratified by treatment arm.
**Fig. S7.** Longitudinal analysis of alpha diversity for patients receiving antibiotics in the first 8 weeks of study treatment.

**Table S1.** Baseline characteristics.
**Table S2.** Univariate Cox regression model for progression‐free survival.
**Table S3.** Multivariate Cox regression model for progression‐free survival.
**Table S4.** Univariate Cox regression model for overall survival.
**Table S5.** Multivariate Cox regression model for overall survival.

## Data Availability

Data are not deposited in a public repository due to data privacy regulations in Norway and our institution. However, data are available upon request if the aims of the planned analyses are covered by the written informed consent signed by the participants, pending an amendment to the ethical approvals and a material and data transfer agreement between the institutions. The transfer of data or materials will require approval from the Data Privacy Officer and Institutional Review Board at OUH, and from the Regional Committee for Medical and Health Research Ethics South‐East Norway and the Belgian Federal Agency for Medicines and Health Products. Any shared data will be de‐identified. Requests should be made to the corresponding author (jonky@ous-hf.no).
