# Supplementary material for: Gut microbiota diversity is prognostic in metastatic hormone receptor‐positive breast cancer patients receiving chemotherapy and immunotherapy
Source: Mol Oncol. 2025 Aug 25;20(2):511–23. doi: 10.1002/1878-0261.70117 (PMC12936421; doi:10.1002/1878-0261.70117)
Supplement: Supplementary file 1 — Fig. S1. Kaplan–Meier plots of progression‐free survival (a) and overall survival (b) for patients included in the microbiota analysis in ICON. Fig. S2. Progression‐free survival by baseline alpha diversity stratified by treatment arm. Fig. S3. Overall survival by baseline alpha diversity stratified by treatment arm. Fig. S4. Associations between alpha diversity and line of metastatic chemotherapy. Fig. S5. Associations between alpha diversity and high‐grade immune‐related adverse events. Fig. S6. Longitudinal analysis of alpha diversity stratified by treatment arm. Fig. S7. Longitudinal analysis of alpha diversity for patients receiving antibiotics in the first 8 weeks of study treatment. [file MOL2-20-511-s001.pdf]

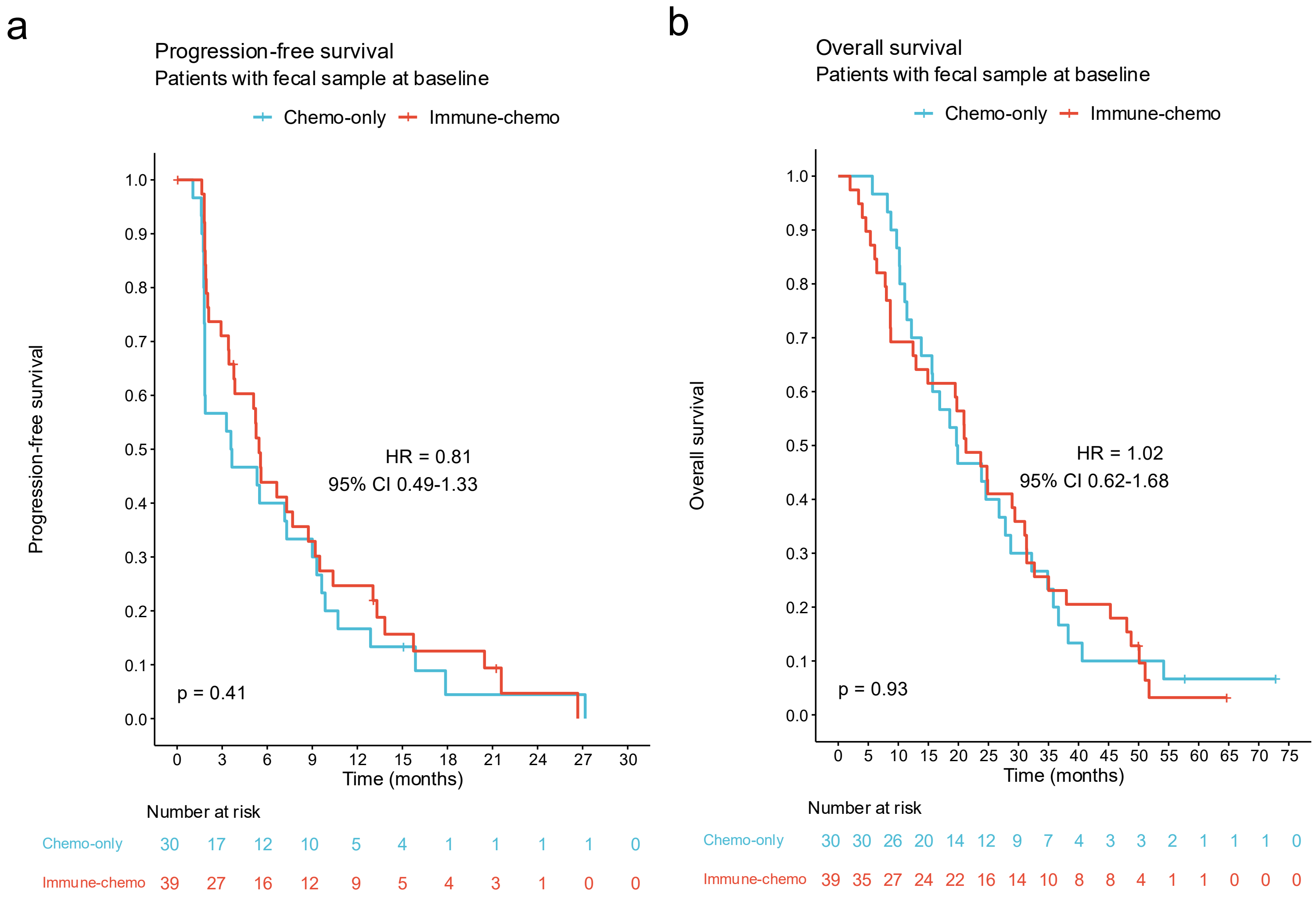

Figure S1.
Kaplan–Meier plots of progression-free survival (a) and overall survival (b) for patients included in the microbiota analysis in ICON.

Hazard ratios with 95% confidence intervals were obtained from the Cox proportional hazards model. *P* values calculated by the log-rank method.

Abbreviations: HR, Hazard ratio; CI, confidence interval.

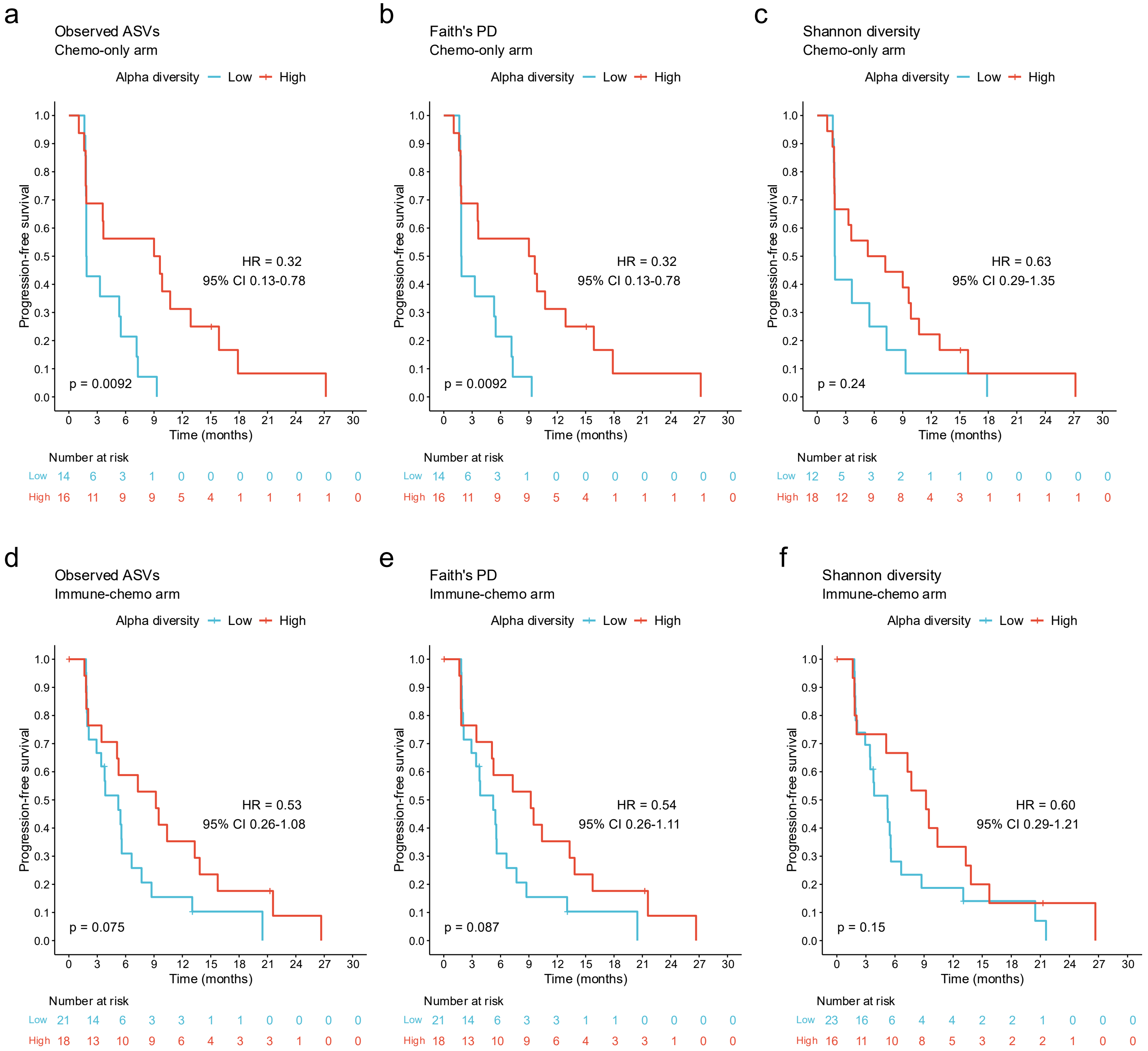

Figure S2.
Progression-free survival by baseline alpha diversity stratified by treatment arm.

(a) Observed ASVs in the chemo-only arm
(b) Faith's PD in the chemo-only arm
(c) Shannon diversity in the chemo-only arm
(d) Observed ASVs in the immune-chemo arm
(e) Faith's PD in the immune-chemo arm
(f) Shannon diversity in the immune-chemo arm

Patient were categorized into low and high diversity groups based on the median score of the alpha diversity metrics. Hazard ratios and 95% confidence intervals were obtained from the Cox proportional hazards model. *P* values were calculated by the log-rank method.

Abbreviations: HR, Hazard ratio; CI, confidence interval; ASV, Amplicon sequence variant; Faith's PD, Faith's phylogenetic diversity

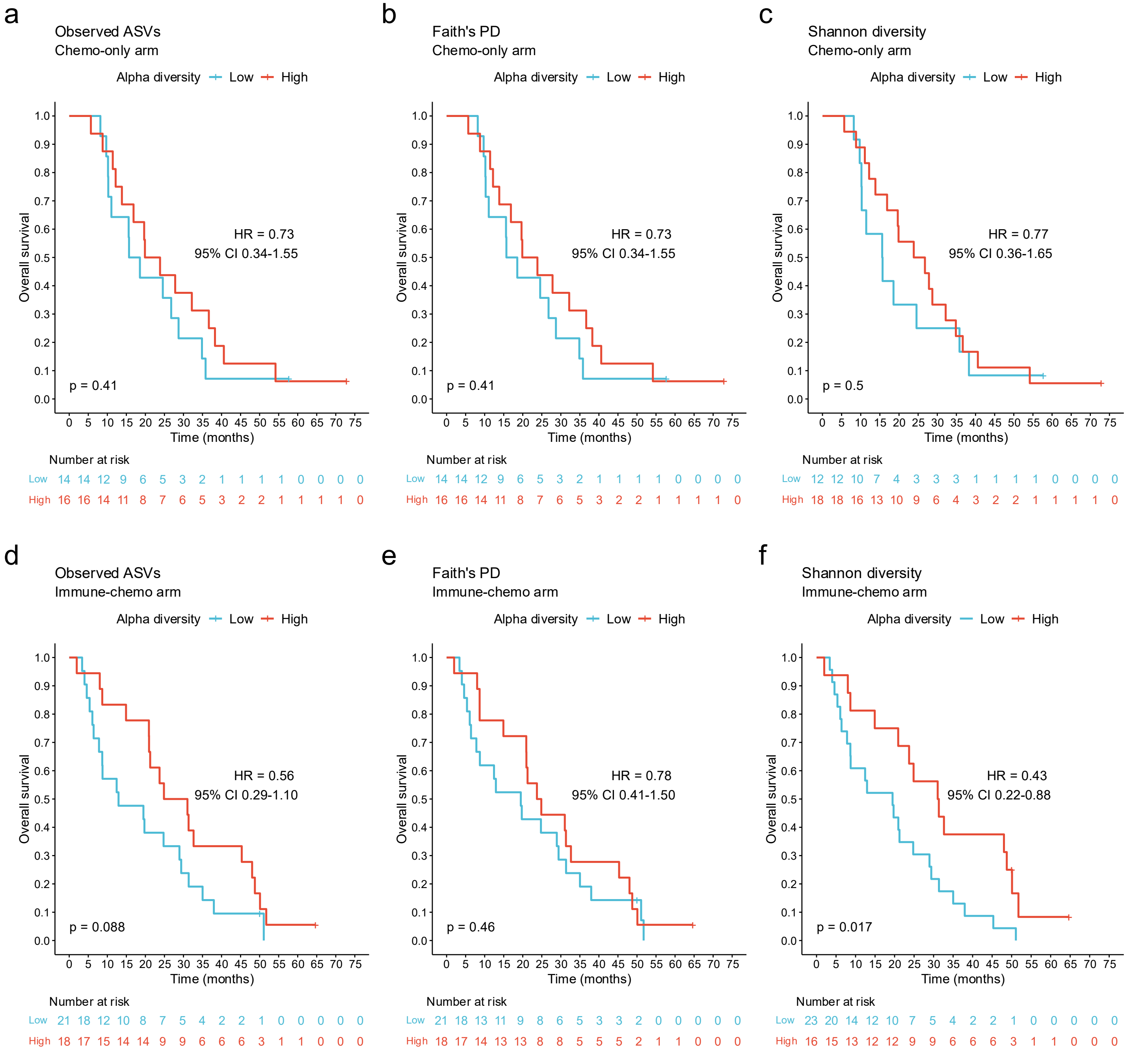

Figure S3.
Overall survival by baseline alpha diversity stratified by treatment arm.

(a) Observed ASVs in the chemo-only arm
(b) Faith's PD in the chemo-only arm
(c) Shannon diversity in the chemo-only arm
(d) Observed ASVs in the immune-chemo arm
(e) Faith's PD in the immune-chemo arm
(f) Shannon diversity in the immune-chemo arm

Patient were categorized into low and high diversity groups based on the median score of the alpha diversity metrics. Hazard ratios with 95% confidence intervals were obtained from the Cox proportional hazards model. *P* values were calculated by the log-rank method.

Abbreviations: HR, Hazard ratio; CI, confidence interval; ASV, Amplicon sequence variant; Faith's PD, Faith's phylogenetic diversity

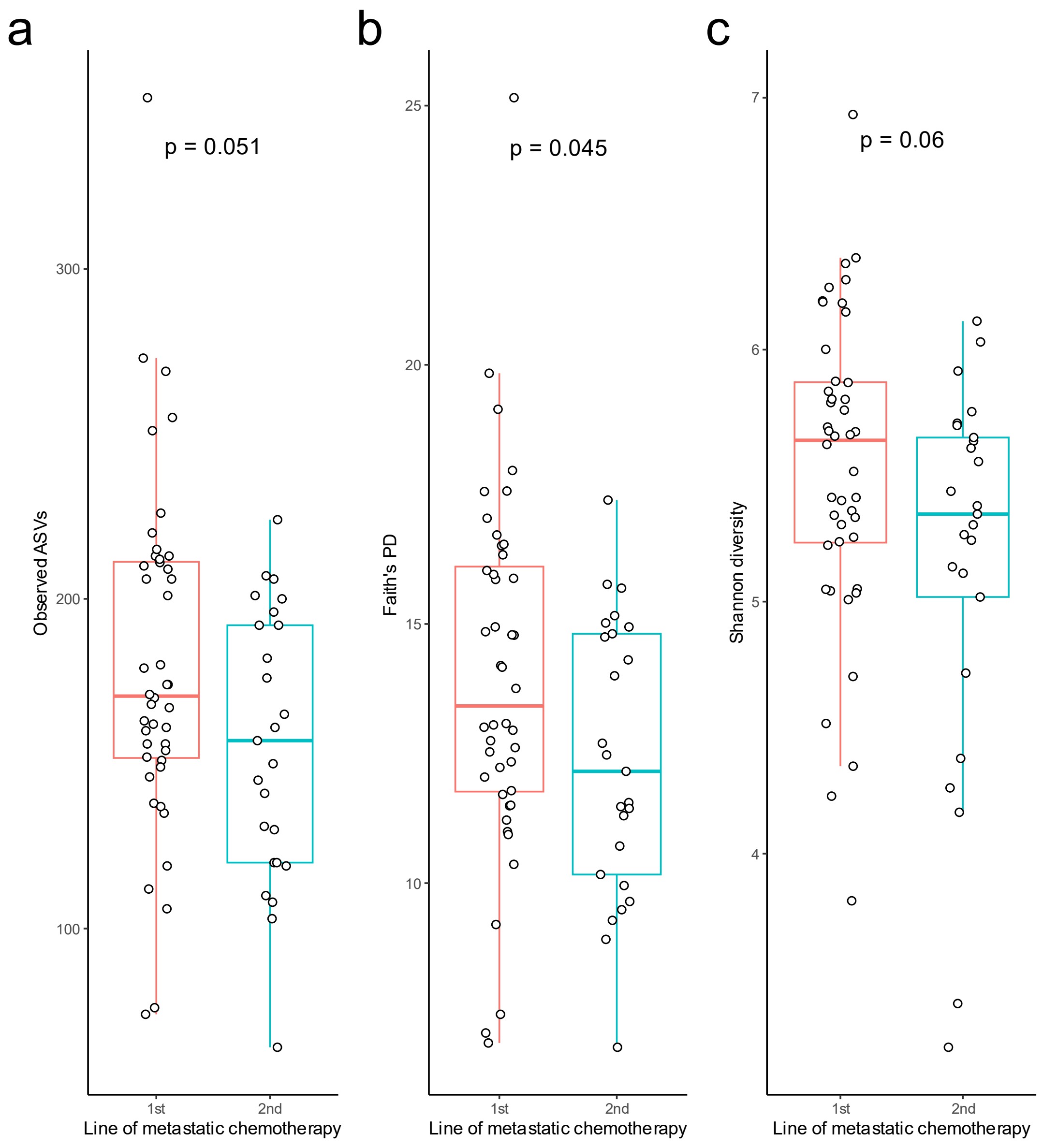

Figure S4. Associations between alpha diversity and line of metastatic chemotherapy.

Alpha diversity in patients with 1st line of metastatic chemotherapy in ICON compared to patients with 2nd line of metastatic chemotherapy in ICON. Different alpha diversity metrics applied a) Observed ASVs (b) Faith's PD and (c) Shannon diversity. *P* values calculated by Wilcoxon rank-sum test. The box plot extends from the first to the third quartile. The middle line represents the median and the whiskers extend to the most extreme points within 1.5 x IQR. Each dot represents a sample.

Abbreviations: ASV, Amplicon sequence variant; Faith's PD, Faith's phylogenetic diversity.

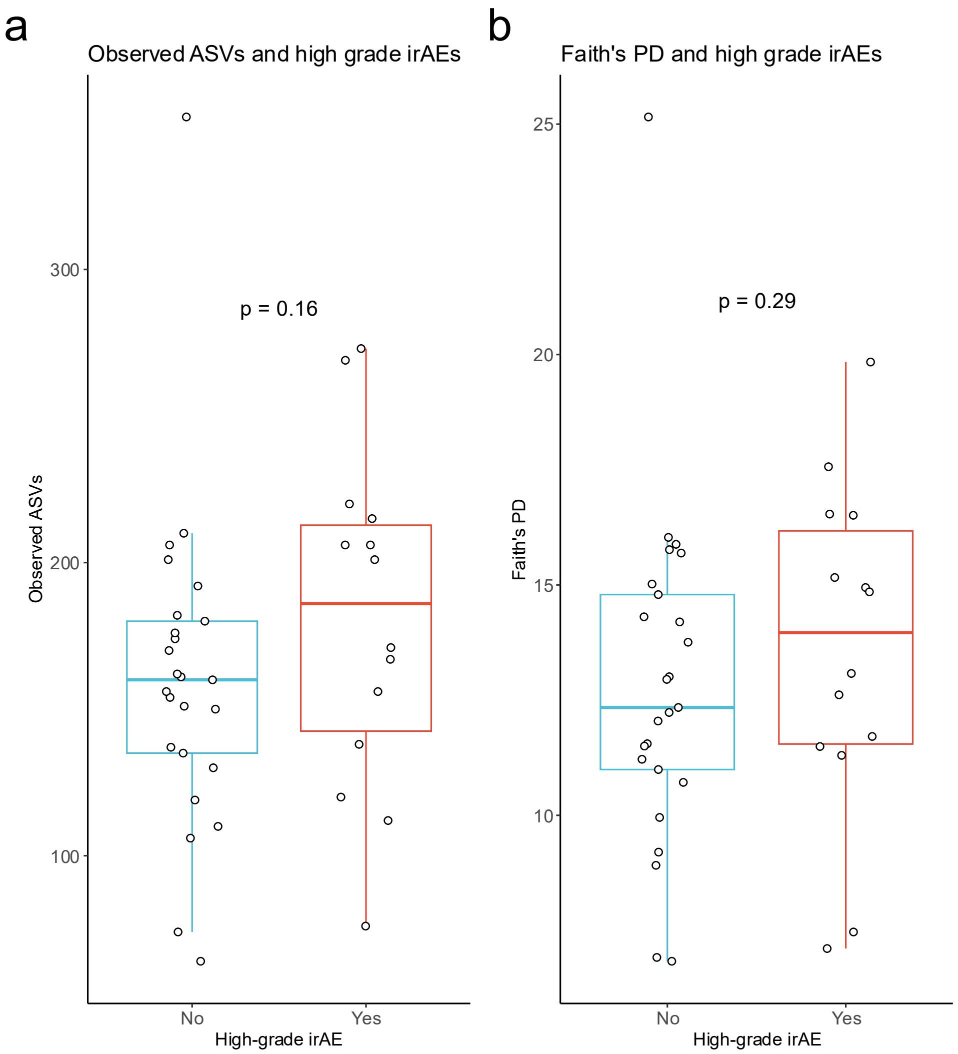

Figure S5. Associations between alpha diversity and high-grade immune-related adverse events.

Observed ASVs (a) and Faith's phylogenetic diversity (b) in patients with and without high-grade irAEs. The box plot extends from the first to the third quartile. The middle line represents the median and the whiskers extend to the most extreme points within 1.5 x IQR. Each dot represents a sample. *P* value calculated by Wilcoxon rank-sum test.

Abbreviations: ASV, Amplicon sequence variant; Faith's PD, Faith's phylogenetic diversity; irAE, immune-related adverse events

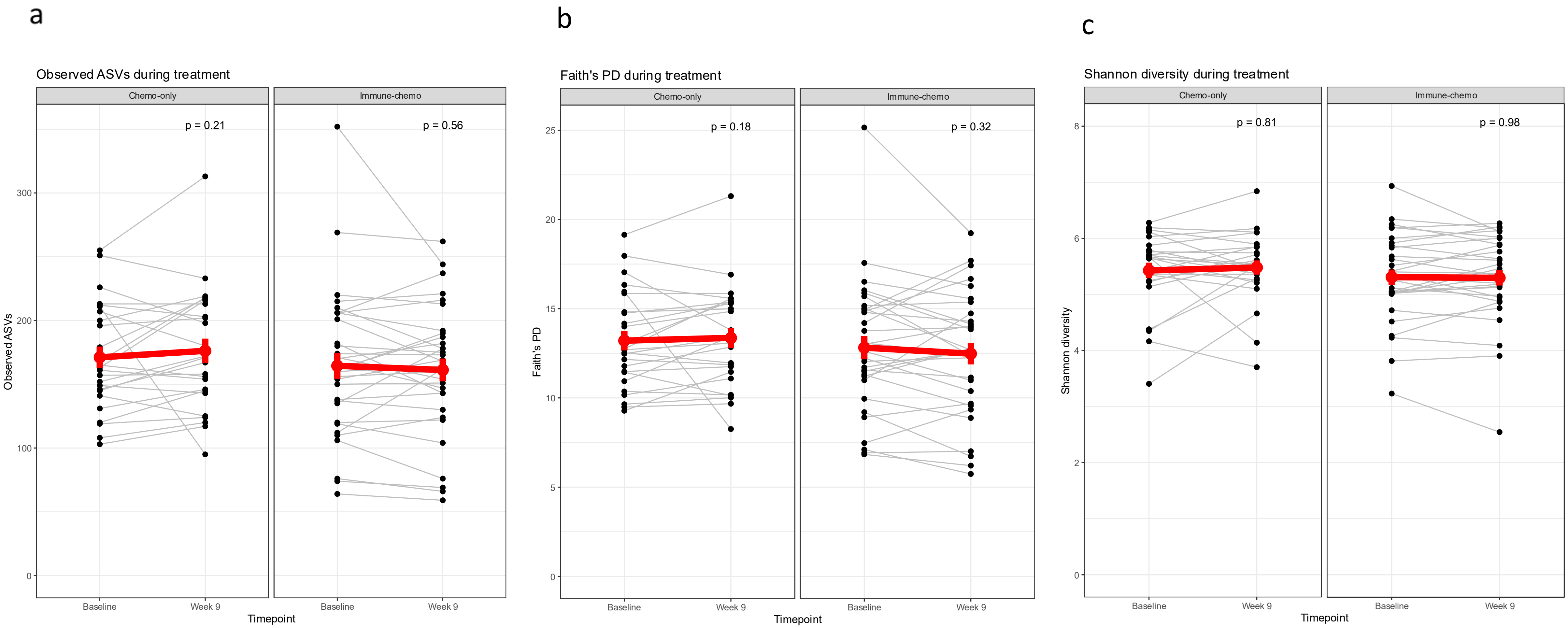

Figure S6. Longitudinal analysis of alpha diversity stratified by treatment arm.

Alpha diversity in the course of study treatment in the two treatment arms.
Different alpha diversity metrics applied a) Observed ASVs (b) Faith's PD and (c) Shannon diversity.
***P* values were calculated using Wilcoxon signed-rank test. The red point represents the mean alpha diveristy at each timepoint and the errorbars represent the standard error of the mean. The red line represents the change of the mean alpha diversity value from baseline to week 9.**

Abbreviations: ASV, Amplicon sequence variant; Faith's PD, Faith's phylogenetic diversity.

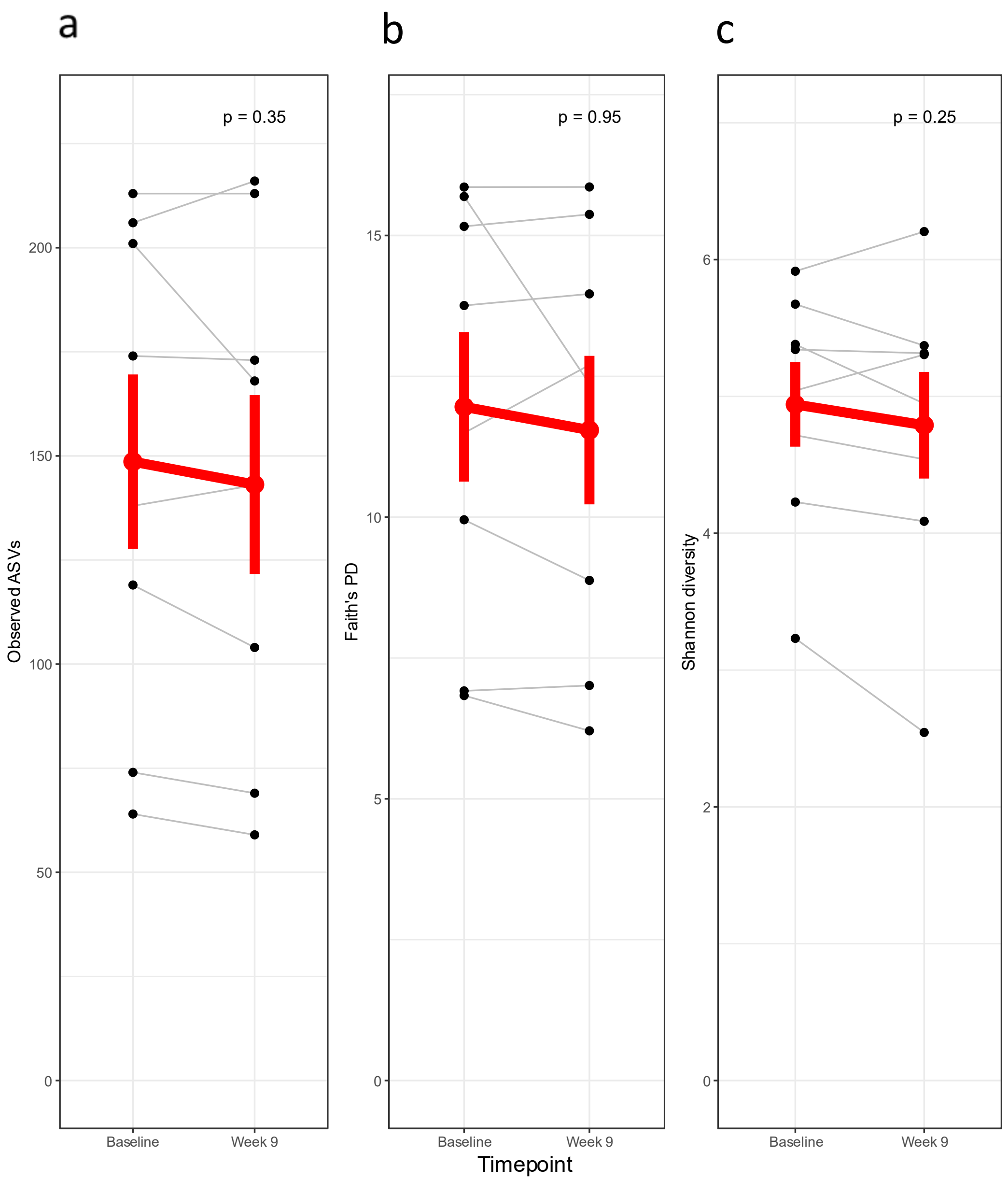

Figure S7. Longitudinal analysis of alpha diversity for patients receiving antibiotics in the first 8 weeks of study treatment.

Eight patients with available paired stool samples (baseline and week 9) received antibiotics during the first 8 weeks in ICON. Different alpha diversity metrics applied a) Observed ASVs (b) Faith's PD and (c) Shannon diversity. *P* values were calculated using Wilcoxon signed-rank test. The red point represents the mean alpha diversity at each timepoint and the errorbars represent the standard error of the mean. The red line represents the change of the mean alpha diversity value from baseline to week 9

Abbreviations: ASV, Amplicon sequence variant; Faith's PD, Faith's phylogenetic diversity.
