# Supplementary material for: Gut microbiota diversity is prognostic in metastatic hormone receptor‐positive breast cancer patients receiving chemotherapy and immunotherapy
Source: Mol Oncol. 2025 Aug 25;20(2):511–23. doi: 10.1002/1878-0261.70117 (PMC12936421; doi:10.1002/1878-0261.70117)
Supplement: Supplementary file 2 — Table S1. Baseline characteristics. Table S2. Univariate Cox regression model for progression‐free survival. Table S3. Multivariate Cox regression model for progression‐free survival. Table S4. Univariate Cox regression model for overall survival. Table S5. Multivariate Cox regression model for overall survival. [file MOL2-20-511-s002.zip › Supplementary Tables.pdf]

Supplementary Tables

	Chemotherapy only<br><br>(N=30)	Chemotherapy plus ipilimumab and nivolumab<br><br>(N=39)	P value
**Age**			0.65
Median [range]	56.0 [37.0, 74.0]	52.0 [36.0, 75.0]	
**ECOG**			0.55
0	16 (53.3%)	18 (46.2%)	
1	14 (46.7%)	21 (53.8%)	
**PAM50 subtype**			0.88
HER-2 enriched	2 (6.7%)	4 (10.3%)	
Luminal A	6 (20.0%)	9 (23.1%)	
Luminal B	19 (63.3%)	24 (61.5%)	
Missing	3 (10.0%)	2 (5.1%)	
**Line of metastatic chemotherapy**			0.11
1st	16 (53.3%)	28 (71.8%)	
2nd	14 (46.7%)	11 (28.2%)	
**Neo-adjuvant chemotherapy**			0.19
No	13 (43.3%)	11 (28.2%)	
Yes	17 (56.7%)	28 (71.8%)	
**Previous CDK4/6 inhibitor**			0.22
No	1 (3.3%)	5 (12.8%)	
Yes	29 (96.7%)	34 (87.2%)	
**Bone metastases**			0.39
No	4 (13.3%)	2 (5.1%)	
Yes	26 (86.7%)	37 (94.9%)	
**Liver metastases**			0.06
No	4 (13.3%)	13 (33.3%)	
Yes	26 (86.7%)	26 (66.7%)	
**Lung metastases**			0.02
No	26 (86.7%)	24 (61.5%)	
Yes	4 (13.3%)	15 (38.5%)	
**>3 sites of metastases**			0.95
No	21 (70.0%)	27 (69.2%)	
Yes	9 (30.0%)	12 (30.8%)	

Table S1. Baseline characteristics.

Two-sided P values were calculated using the Wilcoxon rank-sum test for age and Fisher's exact or chi-square test for categorical data.
Abbreviations: N, number of patients; ECOG, Eastern Cooperative Oncology Group; HER-2, human epidermal growth factor receptor 2; CDK4/6, cyclin-dependent kinase 4 and 6.

Characteristic	N	HR	95% CI	*P* value
Observed ASVs binary	69			
Low		—	—	
High		0.48	0.28-0.81	0.006
Faith's PD binary	69			
Low		—	—	
High		0.48	0.28-0.82	0.008
Shannon binary	69			
Low		—	—	
High		0.65	0.39-1.08	0.094
BMI	69	1.05	0.99-1.11	0.094
Age	69	1.02	1.00-1.05	0.11
ECOG	69			
0		—	—	
1		0.95	0.58, 1.56	0.8
De novo metastatic disease	69			
No		—	—	
Yes		1.36	0.77-2.41	0.3
Previous neodjuvant or adjuvant chemotherapy	69			
No		—	—	
Yes		0.83	0.50-1.40	0.5
Line of chemotherapy	69			
1st		—	—	
2nd		1.93	1.14-3.26	0.014
Number of metastatic sites	69			
≤ 3		—	—	
> 3		1.56	0.90-2.71	0.11
Bone metastases	69			
No		—	—	
Yes		0.39	0.15-1.01	0.053
Liver metastases	69			
No		—	—	
Yes		0.98	0.54-1.79	>0.9
Prior CDK46i	69			
No		—	—	
Yes		0.77	0.33-1.80	0.5
Lines of endocrine therapy in metastatic setting	69			
0		—	—	
1-2		0.78	0.24-2.54	0.7
3		0.61	0.18-2.14	0.4

Table S2. Univariate Cox regression model for progression-free survival.

Univariate Cox proportional hazards analysis for PFS, including all patients in the microbiota analysis from the ICON trial. Low and high diversity groups of alpha diversity were based on the median value.

Abbreviations: N, number of patients; HR, Hazard ratio; CI, confidence interval; ASV, Amplicon sequence variant; Faith's PD, Faith's phylogenetic diversity; ECOG, Eastern Cooperative Oncology Group; CDK4/6, cyclin-dependent kinase 4 and 6.

Covariate	HR (95 % CI)	*P* value
High observed ASVs (unadjusted)	0.48 (0.28-0.81)	0.006
High observed ASVs + Line of chemotherapy	0.49 (0.29-0.85)	0.01
High observed ASVs + Bone metastases	0.49 (0.29-0.84)	0.009
High observed ASVs + BMI	0.50 (0.27-0.92)	0.025
High Faith's PD (unadjusted)	0.48 (0.28-0.82)	0.008
High Faith's PD + Line of chemotherapy	0.50 (0.29-0.86)	0.012
High Faith's PD + Bone metastases	0.50 (0.29-0.85)	0.011
High Faith's PD + BMI	0.51 (0.28-0.94)	0.03

Table S3. Multivariate Cox regression model for progression-free survival.

The hazard ratio and *P* value of progression-free survival for observed ASVs and Faith's PD are shown unadjusted and after adjusting for line of chemotherapy, bone metastases and BMI. Observed ASVs and Faith's PD are treated as binary variables (employing the median value as cutoff). Line of chemotherapy (1st or 2nd) and bone metastases (yes or no) are categorical variables. BMI is a continuous variable.

Abbreviations: HR, Hazard ratio; CI, confidence interval; ASV, Amplicon sequence variant; Faith's PD, Faith's phylogenetic diversity.

**Characteristic**	**N**	**HR**	**95% CI**	***P* value**
Observed ASVs binary	69			
Low		—	—	
High		0.64	0.39-1.05	0.079
Faith's PD binary	69			
Low		—	—	
High		0.75	0.46-1.23	0.3
Shannon binary	69			
Low		—	—	
High		0.59	0.36-0.97	0.036
BMI	69	1.02	0.96-1.08	0.6
Age	69	1	0.97-1.02	0.8
ECOG	69			
0		—	—	
1		1.16	0.71, 1.89	0.5
De novo metastatic disease	69			
No		—	—	
Yes		0.96	0.55-1.69	0.9
Previous neodjuvant or adjuvant chemotherapy	69			
No		—	—	
Yes		1.19	0.71-1.99	0.5
Line of chemotherapy	69			
1st		—	—	
2nd		2.19	1.30-3.69	0.003
Number of metastatic sites	69			
≤ 3		—	—	
> 3		1.57	0.93-2.66	0.095
Bone metastases	69			
No		—	—	
Yes		0.75	0.32-1.76	0.5
Liver metastases	69			
No		—	—	
Yes		0.73	0.42-1.28	0.3
Prior CDK46i	69			
No		—	—	
Yes		0.91	0.39-2.12	0.8
Lines of endocrine therapy in metastatic setting	69			
0		—	—	
1-2		1.33	0.41-4.32	0.6
3		0.68	0.20-2.34	0.5

Table S4. Univariate Cox regression model for overall survival.

Univariate Cox proportional hazards analysis for OS, including all patients in the microbiota analysis from the ICON trial. Low and high diversity groups of alpha diversity were based on the median value.

Abbreviations: N, number of patients; HR, Hazard ratio; CI, confidence interval; ASV, Amplicon sequence variant; Faith's PD, Faith's phylogenetic diversity; ECOG, Eastern Cooperative Oncology Group; CDK4/6, cyclin-dependent kinase 4 and 6.

**Covariate**	**HR (95 % CI)**	***P* value**
High Shannon (unadjusted)	0.59 (0.36-0.97)	0.036
High Shannon + Line of chemotherapy	0.64 (0.39-1.06)	0.082
High Shannon + Number of metastatic sites	0.62 (0.38-1.03)	0.063

Table S5. Multivariate Cox regression model for overall survival.

The hazard ratio and *P* value of overall survival for Shannon diversity are shown unadjusted and after adjusting for line of chemotherapy and number of metastatic sites. Shannon diversity is treated as a binary variable, employing the median value as cutoff. Line of chemotherapy (1st or 2nd) and number of metastatic sites (>3 sites or ≤ 3) are categorical variables.

Abbreviations: HR, Hazard ratio; CI, confidence interval; ASV, Amplicon sequence variant; Faith's PD, Faith's phylogenetic diversity.
